# QTL analysis of flowering time and ripening traits suggests an impact of a genomic region on linkage group 1 in *Vitis*

**DOI:** 10.1007/s00122-014-2310-2

**Published:** 2014-08-12

**Authors:** Iris Fechter, Ludger Hausmann, Eva Zyprian, Margrit Daum, Daniela Holtgräwe, Bernd Weisshaar, Reinhard Töpfer

**Affiliations:** 1 Institute for Grapevine Breeding, Julius Kuehn-Institute, Federal Research Centre for Cultivated Plants, Geilweilerhof, 76833 Siebeldingen, Germany; 2 Faculty of Biology, Center for Biotechnology, Bielefeld University, 33594 Bielefeld, Germany

## Abstract

In the recent past, genetic analyses of grapevine focused mainly on the identification of resistance loci for major diseases such as powdery and downy mildew. Currently, breeding programs make intensive use of these results by applying molecular markers linked to the resistance traits. However, modern genetics also allows to address additional agronomic traits that have considerable impact on the selection of grapevine cultivars. In this study, we have used linkage mapping for the identification and characterization of flowering time and ripening traits in a mapping population from a cross of V3125 (‘Schiava Grossa’ × ‘Riesling’) and the interspecific rootstock cultivar ‘Börner’ (*Vitis riparia* × *Vitis cinerea*). Comparison of the flowering time QTL mapping with data derived from a second independent segregating population identified several common QTLs. Especially a large region on linkage group 1 proved to be of special interest given the genetic divergence of the parents of the two populations. The proximity of the QTL region contains two CONSTANS-like genes. In accordance with data from other plants such as *Arabidopsis thaliana* and *Oryza sativa*, we hypothesize that these genes are major contributors to control the time of flowering in *Vitis.*

**Electronic supplementary material:**

The online version of this article (doi:10.1007/s00122-014-2310-2) contains supplementary material, which is available to authorized users.

## Introduction

Grapevine is a crop used worldwide for fermentation into wine, for fresh and dried fruit supply as well as for juice production. Most grapevine cultivars suffer seriously from various pests and diseases. Thus, a major aim in grapevine breeding is the introgression of traits from *Vitis* species that carry resistances found in species native to Eastern Asia and North America. However, traits such as time of flowering and veraison (beginning of berry ripening) have recently also gained increasing interest due to shifts of plant phenology observed in the context of global warming. The development of molecular markers linked to loci or genes affecting these traits has thus become an important research topic. Such markers will facilitate the early selection of plants on the genetic level, simplifying the introgression of desirable and excluding undesired traits for optimal adaptation of plant development to current climatic conditions.

Several agronomic traits important for grapevine cultivation have been genetically dissected during the past few years. The traits addressed include berry color, berry size and seedlessness (Mejía et al. 2011; Cabezas et al. 2006; Fischer et al. 2004; Doligez et al. 2002), phenology-related traits such as veraison and flowering time (Costantini et al. 2008; Mejía et al. 2007), inflorescence number per shoot (Marguerit et al. 2009) or flower sex (Fechter et al. 2012; Marguerit et al. 2009; Lowe and Walker 2006; Riaz et al. 2004; Dalbó et al. 2000). However, the results usually vary greatly between the genotypes analyzed. Consequently, the identification of loci and genes determining these traits at species level has so far proven difficult. Therefore, the development of robust trait-linked genetic markers remains challenging.

Complex traits that are controlled by many loci such as regulation of flowering time (Costantini et al. 2008) are particularly difficult to analyze, although the published *Vitis* reference genome sequences (Adam-Blondon et al. 2011; Jaillon et al. 2007; Velasco et al. 2007) allow rather easy access to grapevine genes. The genome annotation also enables to compare grapevine genes with genetic data and gene functions assigned in other plants.

Few findings have so far been reported for flowering time control in grapevine. By studying one of the rare mutants known in grapevine, Boss and Thomas (2002) suggested that gibberellic acid inhibits the development of inflorescences. As the authors point out, this is in contradiction with results obtained from model plants such as *Arabidopsis thaliana* where gibberellic acid promotes flowering (Song et al. 2013; Blazquez et al. 1998, Blazquez and Weigel 2000; Langridge 1957). This finding may indicate that greater differences in phenological control mechanisms between annual plants and woody perennial plants like grapevine are to be expected. However, since it seems likely that similar genes are involved, research in *Vitis* has focused on the identification and characterization of candidate genes and gene families for flowering time homologous to those found in *A. thaliana* and other well-studied plants. Using this strategy, the *VvFT*/*TFL1* gene family was already examined in detail and the *VvFT* gene was shown to promote flowering in grapevine as expected from experiments run with model plants (Joly et al. 2004; Boss et al. 2006; Sreekantan and Thomas 2006; Carmona et al. 2007). In addition, the MADS-box gene *VvMADS8* has been characterized in detail (Sreekantan and Thomas 2006). Three members of this subfamily of MADS transcription factor genes are present in the grapevine genome (Carmona et al. 2008). Other work focused on *NAP* (no-apical meristem) homologs from grapevine, being targets of the floral homeotic genes *PISTILLATA*/*APETALA3* in *A. thaliana* (Fernandez et al. 2006). However, compared to the detailed knowledge of the pathways controlling flowering in *A. thaliana* (e.g. Ehrenreich et al. 2009; Andres and Coupland 2012), information about the genes involved in grapevine flowering is still very limited.

The construction of detailed genetic maps in combination with intensive phenotyping and subsequent QTL analysis provides an excellent way to identify genomic regions and define loci involved in expression of the traits investigated. We have chosen this strategy, extended by candidate gene prediction in the target regions, to localize loci controlling flowering and veraison time. The basis for mapping is a cross between the high-quality *Vitis vinifera* breeding line V3125 (‘Schiava Grossa’ × ‘Riesling’) and the interspecific cultivar ‘Börner’ (*Vitis riparia* × *Vitis cinerea*). We searched for flowering time QTLs and closely linked markers using a significantly extended marker set. By analyzing the genotypic and phenotypic segregation in the progeny of V3125 × ‘Börner’, we detected highly significant QTLs on linkage groups 1 and 14. The same QTLs became evident in a second independent mapping population, suggesting an overall role of these regions in the control of flowering time. Genes annotated in the delimited QTL regions include several CONSTANS-like genes as well as other genes that are candidates for a contribution to the control of flowering time.

## Materials and methods

### Plant material

#### Mapping population 1: V3125 × ‘Börner’

The mapping population of 202 individuals was obtained by crossing the cultivars V3125 (‘Schiava Grossa’ × ‘Riesling’) and ‘Börner’ (*V. riparia* Gm183 × *V. cinerea* Arnold) in the years 1998 and 2001 and was planted in the vineyard at Geilweilerhof. V3125 is an elite F1 individual derived from two traditional *Vitis vinifera* cultivars and represents the highly pathogen-susceptible, high-quality *V. vinifera* type. ‘Börner’ is a hybrid of the two American *Vitis* species *V. riparia* and *V. cinerea* and was bred for use as a rootstock cultivar. This cultivar was shown to be highly resistant to different pathogens, including grape phylloxera, downy and powdery mildew and black rot. The F1 progeny of the cross V3125 × ‘Börner’ segregates for several agronomic, morphological and resistance-related traits. Phenotypic and genotypic data of the mapping population were used for the calculation of genetic maps and QTL analysis.

#### Mapping population 2: GF.GA-47-42 × ‘Villard blanc’

This second mapping population was used to confirm selected QTL loci for flowering time found in the V3125 × ‘Börner’ population. It is derived from the cross of the breeding line GF.GA-47-42 (‘Bacchus weiss’ x ‘Seyval’) × ‘Villard blanc’ (Seibel 6468 × ‘Subereux’), comprises 151 F1 individuals and segregates considerably for flowering time. The cross was performed in 1989 and the F1 progeny was planted in the vineyards at Geilweilerhof in 1996. The maternal breeding line GF.GA-47-42 is early flowering. In contrast, ‘Villard blanc’ is an offspring of two late-flowering hybrids and flowers very late. A first genetic map for this mapping population was published in 2006 (Zyprian et al. 2006). This map was improved with additional markers (E. Zyprian, in preparation) and used for QTL analysis.

### Phenotyping of flowering time and veraison

**Fig. 1:**
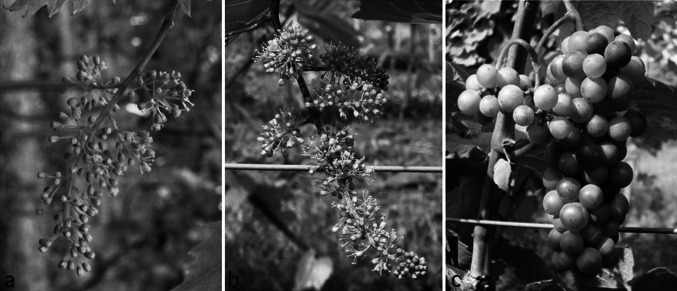
Different developmental stages in the mapping population V3125 × ‘Börner’. **a** first opening of flowers, **b** nearing full bloom, **c** veraison

The progeny of the mapping population V3125 × ‘Börner’ was evaluated in 2010–2012 for flowering time and veraison time (2010–2011) (Fig. 1). Flowering time was scored according to the BBCH scale (Lorenz et al. 1995) as follows: beginning of flowering (stage 61: 10 % of flowerhoods fallen) and full bloom (stage 65: 50 % of flowerhoods fallen). The date at which each individual reached stages 61 and 65 was recorded. The date of veraison time (begin of ripening) was recorded according to the BBCH scale stage 81 (beginning of ripening: berries begin to develop variety-specific color).

The GF.GA-47-42 × ‘Villard blanc’ population was phenotyped for flowering time (full bloom only) in 5 years (1999, 2009–2012). Since these are both white varieties, veraison time was determined as beginning of fruit softening (10–15 % of fruits soft).

For subsequent QTL analysis, the phenotypic data were classified according to five stages for flowering time following OIV descriptor 302 (OIV 2009) (1 = early flowering; 2 = medium early flowering; 3 = medium flowering; 4 = medium late flowering; 5 = late flowering). Assigning the data into these classes proved to be advantageous in comparison to the use of direct dates for QTL analysis as unavoidable differences in flowering dates and periods in the respective years were masked. Using classes thus led to more defined QTL regions with higher LOD scores and smaller confidence intervals. The whole period between the first and last flowering individual was counted in days—starting with the first flowering individual—and divided equally into five periods. Depending on the respective year, each class thus represented 2–4 days of the flowering period. Individuals were then assigned to the according class.

For veraison time, the phenotypic data was classified using a similar system as described above basically following OIV descriptor 303 (OIV 2009). However, it showed that only the application of a 6-class system led to a normal distribution of the data, which—in contrast to the 5-class OIV system—was thus classified to six stages with 1 = very early veraison, 2 = early veraison, 3 = medium veraison, 4 = medium late veraison, 5 = late veraison and 6 = very late veraison.

### DNA extraction

Young leaves were collected and immediately frozen at −70 °C. After lyophilisation of the material, DNA extraction was carried out using the method of Lemke et al. (2011). The DNA was quantified with a spectral NanoPhotometer (Implen GmbH, München, Germany). Samples were adjusted to a DNA concentration of 1 ng/µl for subsequent PCR analyses.

### SSR marker development and analysis

Simple sequence repeat (SSR) markers were designed based on anchored scaffolds from the 8x and 12x version of the published grapevine genome sequence PN40024 internet release (Jaillon et al. 2007; http://www.genoscope.cns.fr/cgi-bin/ggb/vitis/12X/gbrowse/vitis/; Adam-Blondon et al. 2011), respectively. Special focus was laid on regions which had not been covered sufficiently with markers in the previous map elaborated by Zhang et al. (2009). Particular emphasis was laid on the terminal regions of the chromosomes as well as on newly assembled genome regions in the 12x version for which no or only few markers were available before. Supplementary Table S1 gives an overview of all newly designed markers, their source of reference genome (8x or 12x version) and the amplimers. Sequences in regions of interest were scanned for SSRs using the program WebSat (http://wsmartins.net/websat/). Primers flanking the SSRs were developed using the Primer3 software included in the WebSat program. For multiplexing purposes, the length of the amplimers was varied from 80 to 400 bp. Annealing temperatures were set to 60 °C. Forward primers were labeled at the 5′ end with either one of four different fluorescent dyes (HEX, FAM, TAMRA, ROX).

SSR markers were screened for informative segregation using a small subset of genotypes including the parents, grandparents and ten F1 progeny. Additional markers were chosen from literature and also analyzed as described above: VChr- (Cipriani et al. 2008), VMC- (*Vitis* Microsatellite consortium; http://www.agrogene.com), VVMD- (Bowers and Meredith 1996; Bowers et al. 1999), VrZAG- (Sefc et al. 1999) and UDV-(Di Gaspero et al. 2005). All informative markers were combined in multiplexes with 6–12 markers, taking into account the different fragment sizes and fluorescence labels. PCR amplifications were carried out using the Qiagen Multiplex kit (Qiagen GmbH, Hilden, Germany) following the supplier’s instructions. The lengths of the resulting amplicons were analyzed on an ABI 3110xl Genetic Analyzer (Applied Biosystems, Foster City, CA, USA). Marker data and allele calls were collected in flat file format for subsequent analyses.

### Map construction and QTL analysis

For construction of genetic maps, the double pseudo-testcross strategy (Grattapaglia and Sederoff 1994) was applied, resulting in two separate maps for the parents and a consensus map, respectively. The genotypic information was evaluated and the maps were calculated using the program JoinMap 4.0 (Van Ooijen 2006). Markers were tested for their goodness of fit segregation ratio with a Chi-square test. Samples with very high Chi-square values >50 or more than 20 % of missing marker data were excluded from the calculation. Map distances were estimated by applying the Kosambi function (Kosambi 1944). LOD score thresholds of 5 or higher were used for linkage group determination. To avoid map inflation, the reliability of the terminal marker positions was checked with special care by evaluating their data and deduced locations. Suspicious markers that were placed on the map by JoinMap at the outermost positions of the chromosomes with an unreasonable long distance (e.g. 30 or more cM) to all other markers were excluded from the map. In addition, a few markers that were placed at wrong positions with regard to marker phases were also removed from the final dataset, leaving 374 integrated markers.

The data points that had been collected and assigned to the respective phenotypic classes in each season were used for QTL detection. In addition, the median values of the phenotypic classes were also calculated over all years and used as data sets. In our analyses, medians were used instead of means as for some individuals the scored date of flowering time differed greatly in one of the years compared to the other years. Statistically speaking, the median is more robust against these outliers than the mean and therefore represents data that is less skewed. QTL analyses were carried out using the program MapQTL 5.0 (Van Ooijen 2004). Putative QTLs were identified via interval mapping (IM) and subsequent multiple QTL mapping (MQM), using QTL-flanking markers as co-factors. For non-normally distributed traits the non-parametric Kruskal–Wallis rank sum test was applied. Both linkage group specific and genome-wide significance thresholds were determined by permutation tests (1,000 permutations) and the thresholds were set at a value of *p* < 0.05. QTLs were considered reliable within the population if they were identified in at least two growing seasons. Markers associated with peak LOD score values were additionally evaluated for their significance with the Kruskal–Wallis rank sum test.

## Results

### Marker development based on the reference sequence

In total, 215 SSR markers were newly designed based on the reference genome sequence PN40024 (Jaillon et al. 2007; Adam-Blondon et al. 2011) and tested for their segregation type in V3125 × ‘Börner’ (Table 1; supplementary Table S1). Roughly 2/3 (=67 %) of all tested markers could be assigned to one of the segregation types shown in Table 1. Overall, fifteen percent of the markers proved to be monomorphic, while 18 % gave no or unclear signals and were thus excluded from further analysis. 47 % of the usable markers were fully informative segregating with four or three alleles from both parents (ab × cd, ef × eg) and 53 % segregated from only one heterozygous parent (maternal: lm × ll, paternal: nn × np).

**Table 1:** Summary of the markers in the genetic map and their segregation types

	Segregation type	Σ Integrated markers	Newly integrated markers	Integrated markers in total in %	Newly integrated markers in %	% of newly-all integrated markers
Segregating markers included in map		374	191	100	100	51
Fully informative markers (4 resp. 3 alleles)	〈ab×cd〉	168	66	45	35	18
	〈ef×eg〉	32	23	9	12	6
Σ fully informative markers		200	89	53	47	24
Markers heterozygous in only one parent	〈nn×np〉	93	53	25	28	14
	〈lm×ll〉	74	45	20	24	12
Biallelic double heterozygous markers	〈hk×hk〉	7	4	2	2	1
Σ partially informative markers		174	102	47	53	27

### Map construction and extension: the genetic maps

**Fig. 2:**
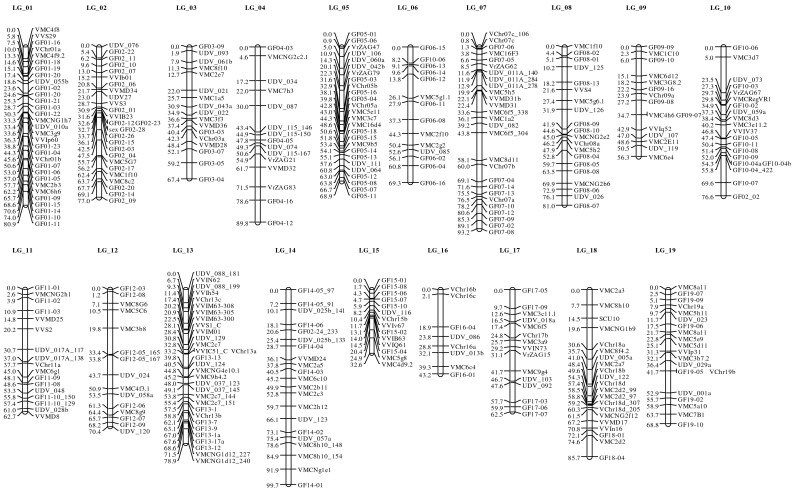
Improved linkage consensus map of the mapping population V3125 × ‘Börner’. Newly developed markers are named GF_linkage group_serial number of marker

One hundred forty-one newly designed markers and 50 additional markers transferred from other public sources (see “Materials and methods”) could be integrated into the previously developed consensus map of V3125 × ‘Börner’ (Zhang et al. 2009). In total, 374 markers could be assigned to 19 linkage groups. These were numbered according to the International Grape Genome Program (IGGP) rules of nomenclature (Adam-Blondon et al. 2004; Riaz et al. 2004). The extended consensus map covered 1365 cM with an average distance of 3.9 cM between the markers (Table 2; Fig. 2).

**Table 2:** Genome length, number of markers per linkage group and average marker distance for the consensus, female and male linkage maps of V3125 × ‘Börner’

LGs	Consensus map			Map of female parent V3125			Map of male parent ‘Börner’		
	Covered length (cM)	No. of markers	Average distance (cM)	Covered length (cM)	No. of markers	Average distance (cM)	Covered length (cM)	No. of markers	Average distance (cM)
1	80.9	31	2.6	72.5	23	3.2	89.7	27	3.3
2	77.0	27	2.9	66.5	13	5.1	72.3	21	3.4
3	67.4	17	4.0	61.3	17	3.6	71.5	16	4.5
4	89.8	15	6.0	56.6	9	6.3	77.1	10	7.7
5	68.9	26	2.7	71.5	21	3.4	79.7	21	3.8
6	69.3	14	5.0	62.2	11	5.7	77.5	10	7.8
7	93.2	27	3.5	100.8	19	5.3	106.5	19	5.6
8	81.0	20	4.1	101.1	20	5.1	84.4	16	5.3
9	56.3	15	3.8	60.4	13	4.6	70.7	9	7.9
10	76.6	20	3.8	53.8	18	3.0	49.1	13	3.8
11	62.7	17	3.7	65.2	11	5.9	63.7	13	4.9
12	70.4	15	4.7	57.1	12	4.8	93.6	13	7.2
13	78.9	31	2.5	72.1	16	4.5	69.1	28	2.5
14	99.7	21	4.7	67.7	17	4.0	107.9	16	6.7
15	32.6	14	2.3	27.9	13	2.1	71.9	12	6.0
16	43.2	8	5.4	43.8	3	14.6	73.7	11	6.7
17	62.5	15	4.2	60.1	12	5.0	66	14	4.7
18	85.7	21	4.1	85.1	15	5.7	107.1	14	7.7
19	68.8	20	3.4	94.4	16	5.9	84.1	15	5.6
*O* (G)	1,364.9	374	3.9	1,280.1	279	5.1	1,515.6	298	5.5

*O* observed genome length

A high degree of co-linearity could be observed between the marker order given in the PN40024 reference genome sequence and the new map presented here. Almost all of the newly designed markers could be assigned to the targeted chromosome region. Only three markers did not localize in the region they were designed from, namely marker GF10-06 (expected on LG10, localized on LG 06), GF02-02 (expected on LG02, localized on LG 10) and GF02-24 (expected on LG02, mapped on LGs 14 and 16).

Using marker development based on the newly assembled and other chromosome parts in the 12x version of the reference genome sequence from PN40024, the map could be extended by 209 cM. Extension of map coverage was achieved particularly at chromosome ends. It should be noted that the percentage of newly identified informative SSR markers varied strongly for the different chromosomes. It proved especially difficult to develop markers for chromosome 16, on which only 20 % of all tested markers showed informative segregation. In addition, only one of them, namely GF16-01, was fully informative while the other markers on this linkage group were segregating only from one (heterozygous) parent yielding limited information.

### Phenotypic data

**Fig. 3:**
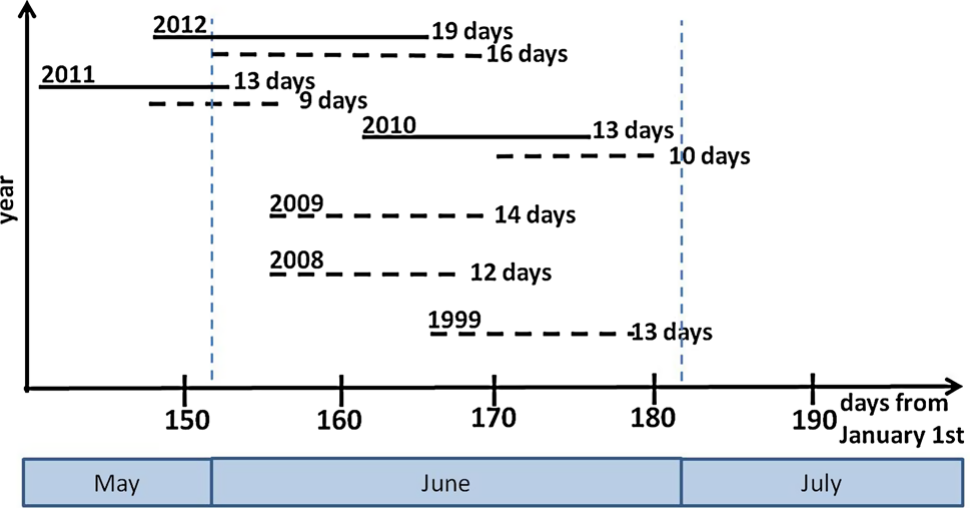
Time span between the first and last genotype reaching full bloom in the mapping population V3125 × ‘Börner’ (*black line*) and GF.GA-47-42 × ‘Villard blanc’ (*dashed line*)

**Fig. 4:**
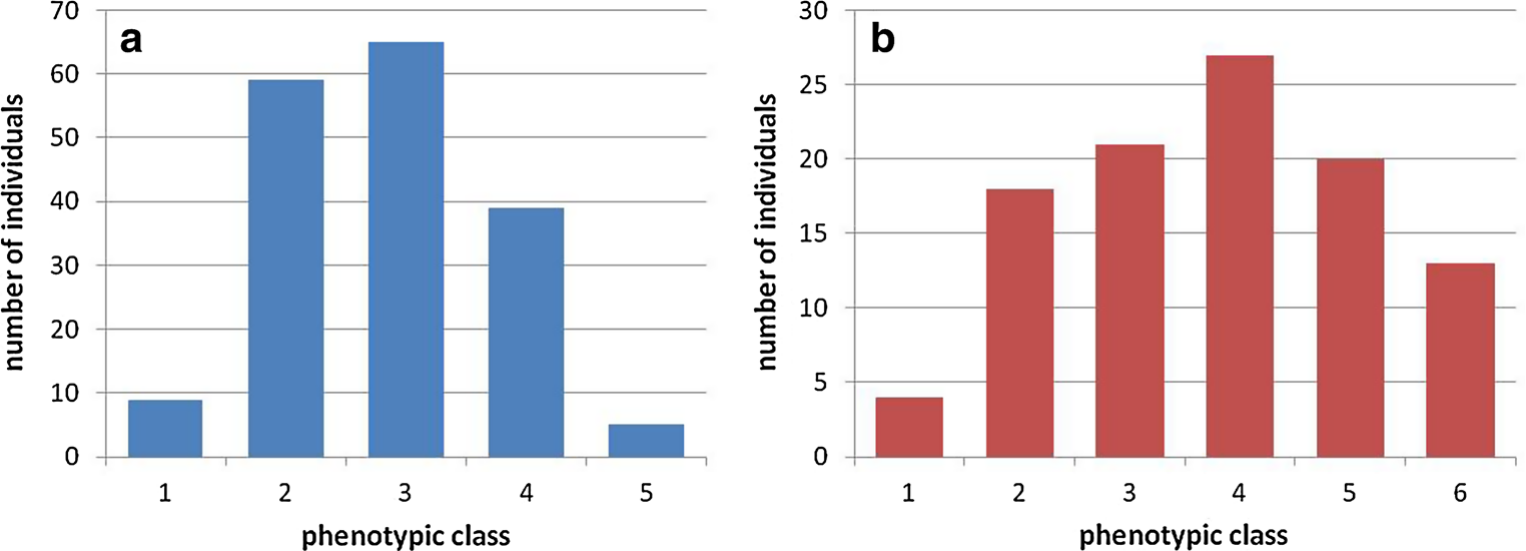
Distribution of classified phenotypic data (median values over the years 2010–2012) in the mapping population V3125 × ‘Börner’. **a** full bloom, **b** veraison time

Flowering time variation in the mapping population V3125 × ‘Börner’ was recorded twice per season, firstly at the onset of opening of flowers and a second time at full bloom. Begin of flowering and full bloom started at different time points between May and June in the years 2010–2012, with an interval of 2–4 days between begin of flowering and full bloom (data not shown). The time interval until all genotypes had reached the full bloom stage also varied between 13 days in the years 2010/2011 and 19 days in 2012 (Fig. 3). The respective data correlated with *r* = 0.4–0.5 (Pearson’s correlation coefficient) between all the years. The frequency distribution of full bloom time over all years is presented in Fig. 4a.

Veraison time (begin of berry ripening) was recorded 39–79 days after full bloom for the mapping population V3125 × ‘Börner’. The data correlated with *r* = 0.3 between the years 2010–2011 and 2011–2012 and *r* = 0.5 between years 2010 and 2012. The frequency distribution over all years is presented in Fig. 4b.

The progeny of the mapping population GF.GA-47-42 × ‘Villard Blanc’ was phenotyped for full bloom only. In this population, flowering started 3–8 days later than in the first mapping population V3125 × ‘Börner’ in the respective years (Fig. 3).

### QTL mapping for flowering time and ripening traits

Using the extended genetic map and the phenotypic data captured from the 202 genotypes in the V3125 × ‘Börner’ population, a detailed QTL analysis was performed. Several QTLs were detected for the agronomic traits considered. Flanking markers of primarily identified major QTLs were used as co-factors for subsequent MQM mapping. Few major QTLs that explain more than 20 % of the phenotypic variance were identified. In addition, a number of weaker QTLs were detected that varied in their position and relevance throughout the years. These “smaller” QTLs were only considered reliable if they were detected in at least two or more different seasons. The phenotypic data did not always exhibit a normal distribution. Additional Kruskal–Wallis tests were thus carried out for evaluation of the QTL results. An overview of the results of all QTL analyses based on the median values of the data sets is given in Table 3. The segregation pattern of the respective trait is indicated in regard to its parental origin.

**Table 3:** Results of the QTL analysis for flowering/ripening-related traits in the mapping population V3125 × ‘Börner’ using the median data sets

Flowering/ripening traits	LG	Parental effect	QTL position (cM)	Confidence interval ± 1 LOD in cM	LOD peak	LOD threshold *a* = 0.05	Marker	% variation explained	KW sign
Start of flowering (*fl*-*s*)	1	p	10.8	5.5–17.3	4.4	3.0	VChr01a_224	10.3	–
	1	p	30.6	27.3–32.1	5.9	3.0	GF01-22	14.3	7
	10	p	54.3	50.9–62.3	3.1	2.8	GF10-04b	7.8	6
	11	p	20.7	15.3–29.7	2.7	2.7	VVS2	6.7	3
	14	p	37.8	31.7–47.7	3.7	3.0	VMC2a5	8.6	7
	14	m	64.7	57.8–75.9	3.6	3.0	UDV_123	9.3	4
	17	m	36.1	21.4–47.6	3.4	2.7	(−)	9.4	–
	17	m	55.1	47.6–62.4	2.9	2.7	GF17-03	7.6	5
Time of full bloom (*fl*-*bl*)	1	p	16.3	11.3–18.5	5.0	3.0	GF01-19	12.8	7
	1	p	30.6	28.8–32.1	5.6	3.0	GF01-22	12.7	7
	10	p	54.3	39.9–64.8	3.0	2.7	GF10-04b	7.2	5
	11	p	20.2	16.3–27.2	4.1	3.0	VVS2	9.4	5
	14	m	61.2	56.3–66.1	3.6	2.9	VMC2h12	9.0	4
	14	m	75.4	66.1–84.6	3.4	2.9	UDV_057a	10.0	1
	16	mp	21.9	19.4–27.3	4.9	2.4	UDV_086	28.9	1
	17	m	35.6	23.9–47.6	3.5	2.7	(−)	10.0	–
	17	m	52.6	47.6–59.2	3.1	2.7	UDV_092	8.5	1
	19	p	34.1	25.6–45.2	3.4	2.8	VMC3b7.2	8.6	3
Veraison (*ver*)	1	p	15.8	9.4–18.7	4.2	3.1	GF01-19	17.7	7
	1	p	30.6	25.8–35.3	5.4	3.1	GF01-22	19.7	7
	11	p	20.2	15.8–24.7	4.2	2.8	VVS2	15.6	5
	13	p	57.5	55.9–59.8	3.8	3.0	GF13-1	14.4	5

All QTLs shown are reproducible over at least 2 years

*LG* linkage group, parental effect (*p* paternal, *m* maternal, *mp* both maternal and paternal), *KW* *sign* Kruskal–Wallis significance level (*p* values) with 1 = 0.1, 2 = 0.05, 3 = 0.01, 4 = 0.005, 5 = 0.001, 6 = 0.0005, 7 = 0.0001, *cM* centiMorgan, *LOD threshold* linkage group specific LOD threshold

For the start of flowering, genomic regions showing significant effects were detected on chromosomes 1, 10, 11, 14 and 17 (see Table 3; marked *fl*-*s*). The same positions were determined for factors affecting the time of full bloom (*fl*-*bl*). Additional reliable QTLs for time of full bloom were detected on linkage groups 16 and 19. However, the QTL on linkage group 1 showed the most consistent effects over all years and explained 12.7–12.8 % of phenotypic variance. The 1-LOD interval for the time of full bloom spanned 7.2 cM around the markers GF01-19 and GF01-20. A second LOD peak with a confidence interval of 3.3 and 4.8 cM is found for time of full bloom and the beginning of flowering, respectively, with the same corresponding marker GF01-22. The large QTL region on chromosome 1 covers an area of 20.8 cM for full bloom. Referring to the reference genome sequence PN40024, the physical location for the QTL is between 2.8 and 8.2 Mb. An additional two-peak QTL was detected for the beginning of flowering on LG 14 with the associated markers VMC2a5 and UDV_123. Searching genetic factors affecting full bloom, two adjacent loci on the same chromosome were detected. They stretch over a 1-LOD confidence interval of 9.8 respective 18.5 cM with the corresponding markers VMC2H12 and UDV_057a.

**Fig. 5:**
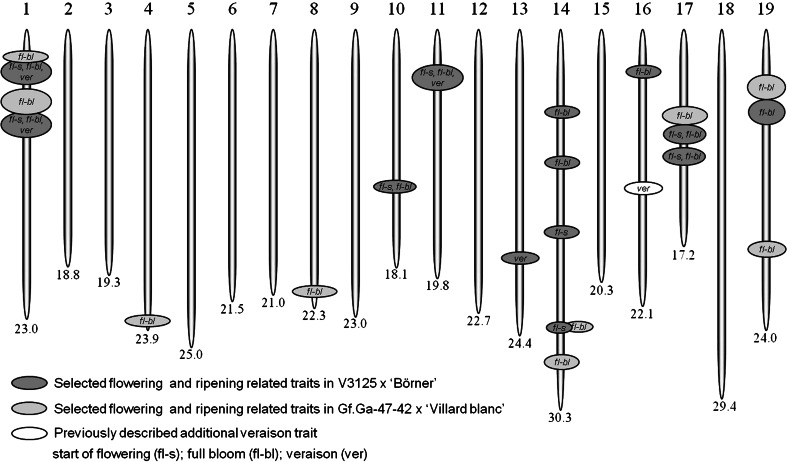
Graphical scheme of the 19 chromosomes of *Vitis*. Indicated are the physical locations of the flowering and ripening-related traits from the mapping population V3125 × ‘Börner’ and GF.GA-47-42 × ‘Villard blanc’, respectively. An additional veraison QTL that has been described previously is depicted on chromosome 16 (Fischer et al. 2004; Costantini et al. 2008)

Several regions relevant to determine veraison time were detected on chromosomes 1, 11 and 13 (Table 3; marked *ver*). The QTLs on chromosomes 1 and 11 covered the same genomic regions as those for full bloom. The QTL on chromosome 1 explained 17.7 % resp. 19.7 % of the phenotypic variance. While the QTL region exceeding the significance threshold was extending over 31.3 cM and showed three distinct peaks in 2010, the 1-LOD interval was drastically reduced to 3.4 cM in the following year with marker GF01-19 lying closest to this QTL. Altogether, the same QTL was identified for veraison time and for flowering time. For the flowering–veraison interval, no significant effects could be determined. All results concerning flowering time and ripening-related traits in the mapping population of V3125 × ‘Börner’ are summarized in Fig. 5.

For the second mapping population GF.GA-47-42 × ‘Villard Blanc’, QTLs for flowering time (full bloom) were detected on the same chromosomes 1, 14, 17 and 19. In addition, further QTLs were located on chromosomes 4 and 8 (Table 4).

**Table 4:** Results of the QTL analyses for full bloom in the mapping population GF.GA-47-42 × ‘Villard Blanc’ using the median data sets

Flowering trait	LG	Parental effect	QTL position (cM)	Confidence interval ± 1 LOD in cM	LOD peak	LOD threshold *a* = 0.05	Marker	% variation explained	KW sign
Time of full bloom (*fl*-*bl*)	1	m	11.0	4.6–15.0	4.2	3.1	GF01-16	17.0	–
	1	m	27.9	16.7–29.9	4.5	3.1	VRZAG29	14.6	4
	4	m	3.6	1.0–7.6	5.1	3.2	VMC6G10	16.5	6
	8	m	3.6	0.0–23.2	4.3	3.1	SNP1295	31.2	–
	14	m	56.7	55.6–58.4	6.2	3.8	VMC2A5	21.1	6
	14	m	65.2	60.4–70.5	5.6	3.8	UDV_095	18.4	7
	17	m	24.0	18.0–28.5	3.9	2.7	VRZAG15	15.9	3
	19	m	16.8	10.6–31.1	3.5	2.8	UDV_127	11.9	–
	19	m	39.2	35.4–42.2	3.4	2.8	VMC5E9	11.3	4

All QTLs shown are reproducible over at least 2 years

*LG* linkage group, parental effect (*p* paternal, *m* maternal), *KW sign* Kruskal–Wallis significance level (*p* values) with 1 = 0.1, 2 = 0.05, 3 = 0.01, 4 = 0.005, 5 = 0.001, 6 = 0.0005, 7 = 0.0001, *cM* centiMorgan, *LOD threshold* linkage group specific LOD threshold

For chromosome 1, two QTL peaks could be determined with the corresponding markers GF01-16 and VrZAG29. They explain 17.0 respective 14.6 % of the phenotypic variance. The physical positions of the flanking markers of their 1-LOD confidence intervals indicate that these regions are located between 3.46 and 5.98 Mb with the associated marker VrZAG29 at a position of 5.28 Mb. The two regions overlap to a great extend with the large QTL region on the same chromosome found in the mapping population V3125 × ‘Börner’ (2.8–8.2 Mb).

On LG 14, the QTL around marker VMC2A5 explains 21.1 % of the phenotypic variance for the time of full bloom. The same marker was found to be closest to the QTL peak for the start of flowering in the mapping population V3125 × ‘Börner’ but remained undetected for the time of full bloom. An overview of all QTLs identified in the mapping population GF.GA-47-42 × ‘Villard blanc’ is given in Table 4.

## Discussion

In the recent past, genetic analyses have led to the identification of numerous resistance loci for the most important grapevine diseases powdery and downy mildew (for review see Töpfer et al. 2011). These loci have been characterized, and some of them are used via marker-assisted selection (MAS) in breeding programs. Despite the fact that there is further work required to optimize markers for durable resistance, breeders also demand for molecular markers addressing other agronomic and resistance-related traits to support breeding. Up to now, there are only few suitable markers available for genetically well-described quantitative or polygenic agronomic traits. QTL mapping is one of the most successful approaches for trait-linked marker development. We have thus evaluated mapping populations for phenological traits (flowering time, veraison) using this method. The results were compared with the outcomes of other research groups trying to identify QTLs which can reliably be characterized in different genetic backgrounds.

### Map construction

The published genome sequence of the nearly homozygous inbred line PN40024 (http://www.genoscope.cns.fr/externe/GenomeBrowser/Vitis/) was used for SSR marker development to increase saturation in the genetic map of the interspecific cultivar ‘Börner’, a hybrid of *V. riparia* × *V. cinerea*.

Testing the newly deduced markers showed that an unexpectedly high amount of 82 % was amplification-positive in pretests and two-thirds were informative in segregation analysis. Moreover, most of the newly designed markers could be assigned to the targeted chromosome regions, which confirms the high synteny within the genus *Vitis* and the usefulness and reliability of the reference genome sequence for marker design. Genomes of different *Vitis* species have been shown to have a high degree of variability with an average of 3.4 million SNPs (1 every 140 bp) per genotype species (Le Paslier et al. 2013). We therefore expected a much higher rate of amplification failure than observed, especially as this work was carried out with a progeny of an interspecific cross of the type *V. vinifera* × (*V. riparia* × *V. cinerea*). Some of the linkage groups, however, proved difficult to be covered with new markers. Especially linkage group 16, on which only 20 % of all tested markers showed segregation, could not be covered as satisfactorily as the other linkage groups. Segregation was only detectable from the male parent, giving rise to only partially informative markers. As the mapping population genetically represents three different *Vitis* species, these results could be due to altered levels of heterozygosity in the corresponding chromosome or chromosomal segments. Chromosome 16 seems to be highly homozygous in *V. vinifera* and thus only shows a few detectable length polymorphisms for the two American species of the ‘Börner’ genome. In addition, chromosome 16 seems to contain large stretches of repetitive sequences, making it difficult to design unique markers. However, the overall results and statistics prove that the strategy of designing SSR marker assays (primer sequences) from an already existing reference sequence is a powerful and easy-to-use tool for future marker design.

### Flowering and ripening-related parameters

**Fig. 6:**
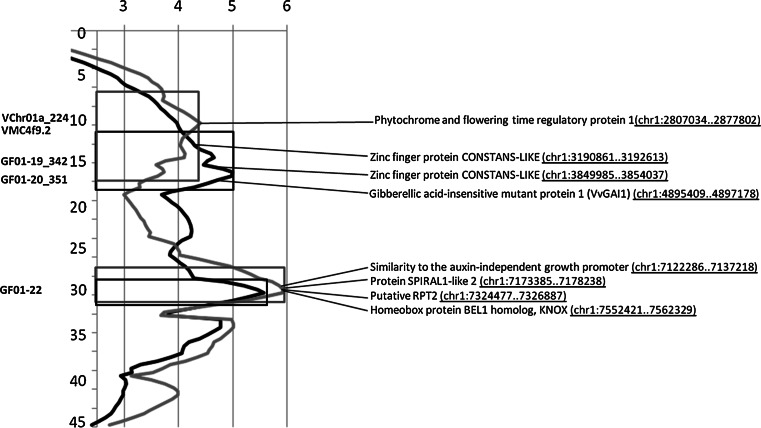
QTL region on chromosome 1 in the mapping population V3125 × ‘Börner’ for start of flowering (*light green line*) and full bloom (*dark red line*) and relative location of annotated candidate genes in the reference genome sequence; *Y*-axis in cM. Correlating marker positions are shown on the *left*. The positions of candidate genes in the physical map of the reference sequence PN40024 (12x) are indicated in *brackets* behind the putative candidate genes. *Rectangles* indicate the 1-LOD interval for each peak (color figure online)

The most consistent QTL in this study was a region on LG 1 affecting both flowering and veraison time, with two distinct peaks which together cover a broad confidence interval of 20.8 cM respective 5.4 Mb of the chromosome in the first mapping population V3125 × ‘Börner’ (Fig. 6). QTL analyses in the second segregating population of GF.GA-47-42 × ‘Villard blanc’ indicated that especially this region on chromosome 1 might be of interest regarding different phenology traits including the time of full bloom. For this developmental stage, the same QTL regions on chromosome 1 were found independently in both mapping populations, using different sets of markers and phenotypic data.

QTLs for flowering and veraison time and the flowering–veraison interval were also previously reported for chromosomes 1 and 2 by Costantini et al. (2008). Their closest marker linked to flowering time is located approximately 6 Mb away from the QTL detected in this study. In the mapping population V3125 × ‘Börner’, we could not identify reproducible significant QTLs for the flowering–veraison interval. The reason for this might be due to the high consistency between flowering and veraison time QTLs in this mapping population as early-flowering plants also reached veraison time earlier than the late-flowering plants. Just one additional chromosome, namely chromosome 13, was found to be only related with veraison time in comparison to the time of flowering, meaning that there are almost no differences in the intervals between the two traits which could be mapped as a QTL.

Additional QTLs for flowering time were observed on chromosomes 10, 11, 14, 16, 17 and 19 in the mapping population V3125 × ‘Börner’ (Fig. 5), underlining the hypothesis of a complex trait with numerous genes involved in flower formation and the onset of flowering. The effects on chromosome 14 and 17 were also evident in the second mapping population. On both of these LGs, loci have already been proposed for the flowering time trait (Duchene et al. 2012; Mejía et al. 2007). The QTL on LG14 identified by Duchene et al. (2012) is located in close proximity to the one we found in this study. The authors propose the gene *VvCOL2* as a candidate gene for flowering time. All results indicate that a combination of several genomic regions influences flowering parameters, with each of them contributing only a more or less small effect to the overall performance of the plants.

The difference in flowering time (early vs. late) is inherited from different genetic backgrounds in the two mapping populations, leading to the shared QTLs shown in Tables 3 and 4. For the progeny of V3125 × ‘Börner’, genetic factors inherited from *V. cinerea* contributed most to the late-flowering phenotypes on LG 1, 10, 11 and 19 while the alleles from *V. riparia* led to early flowering. For the QTLs on LG 14 and 17, effects were observed for ‘Schiava Grossa’ (early flowering) and ‘Riesling’ (late flowering) (Table 3). In the pedigree of the second mapping population GF.GA-47-42 × ‘Villard blanc’, however, early flowering was exclusively transmitted by the ancestors of GF.GA-47-42, suggesting that early flowering is a dominant effect. Interspecific French hybrids are the origin of the late-flowering ‘Villard blanc’ (Seibel 6468 and ‘Subereux’). These include *V. rupestris* in their pedigrees. Although genetically unrelated, the alleles inherited from the parents of the two mapping populations led to the same effect of early respective late flowering (cf. “Materials and methods” section “Plant material”).

In regard to the control of flowering time, complex mechanisms have been revealed in model plants such as *A. thaliana* (e.g. Ehrenreich et al. 2009), giving a hint at genes that might also contribute to flowering and ripening time control in *Vitis* species. With the availability of a reference genome sequence for grapevine, it has become possible to postulate candidate genes homologous to *A. thaliana* or other plant flowering time control genes in the grapevine genome. These include flowering integrator genes such as members of the FT/TFL1 family (Joly et al. 2004; Boss et al. 2006; Carmona et al. 2007; Sreekantan and Thomas 2006) and flower meristem identity genes, e.g. transcription factor LEAFY (VFY in grapevine) (Carmona et al. 2002; Joly et al. 2004; Boss et al. 2006) or the MADS-box genes APELATA1 (VAP1) and FRUITFULL (VFUL-L) (Calonje et al. 2004). Based on the reference sequence annotation, several genes homologous to known plant genes—especially from *A.* *thaliana* and *O. sativa—*are located within the 1-LOD confidence interval of the QTL on chromosome 1 that might contribute to flowering time (Tables 5, 6; Fig. 5). Directly at the QTL peak for the start of flowering around marker VChr01a, the *phytochrome and flowering time regulatory protein 1* (PFT1) is annotated in the reference sequence (Fig. 6). It therefore represents an important candidate gene for flowering time control. As a subunit of the plant mediator complex, the most important targets of PFT1 concerning flowering time control have been shown to be the important flowering genes CONSTANS (CO) and Flowering Locus T (FT). PFT1 serves as an independent transcriptional activator of both these genes (Iñigo et al. 2012). The QTL LOD value is highest around the region of *VvPFT1* for the onset of flowering. VvPFT1 may initiate a pathway leading to the activation of CO and FT, subsequently followed by the action of other genes.

**Table 5:** Selected candidate genes annotated in the reference genome sequence of PN40024 for the flowering time QTL around markers VChr01a-GF01-19 on chromosome 1

Marker	Physical location	Location in cM	Candidate gene	Location of gene on physical map	Proposed function(s)
			AP2/ERF and B3 domain-containing transcription repressor RAV2	(chr1:2751757..2752602)	Ethylene-responsive transcription factor RELATED TO APETALA2 8 *Arabidopsis thaliana.* Transcriptional repressor of flowering time on long day plants. Acts directly on FT expression
VChr01a_224	2,819,297	10.758	Phytochrome and flowering time regulatory protein 1	(chr1:2807034..2877802)	Acts in repression of PhyB-mediated light signaling and regulates the expression of FLOWERING LOCUS T (FT) and of CONSTANS (CO)
			Transcription factor PERIANTHIA	(chr1:2941875..2946272)	Transcriptional activator involved in the determination of floral organ number. Acts to determine floral organ patterning by establishing floral organ primordia in specific numbers and positions
VMC4f9.2	3,064,237	13.478			
			AP2/ERF transcription factor family	(chr1:3143063..3143599)	Berry development
			Zinc finger protein CONSTANS-LIKE	(chr1:3190861..3192613)	Regulation of FT expression
GF01-18	2,997,334	14.639			
			AS2/LOB domain gene family	(chr1:3210859..3211373)	Differentiation of floral whorls
			Zinc finger protein JAGGED	(chr1:3253445..3255253)	Another development; carpel development; leaf morphogenesis; specification of floral organ identity
GF01-19_342	3,794,270	15.332	Auxin-responsive protein	(chr1:3720088..3721588)	Plant growth and development
			Zinc finger protein CONSTANS-LIKE	(chr1:3849985..3854037)	
			Homeodomain transcription factor HOX17	(chr1:4488307..4490678)	Cell proliferation
			Ripening-related protein	(chr1:4747886..4748614)	
			Ripening-related protein	(chr1:4757268..4775296)	
			Ripening-related protein	(chr1:4774561..4775296)	
			Ripening-related protein	(chr1:4810650..4821138)	
			Senescence related protein	(chr1:4879232..4879778)	
			*Gibberellic acid*-*insensitive mutant protein 1;*VvGAI1	(*chr1:4895409..4897178*)	Probable transcriptional regulator that acts as a repressor of the gibberellin (GA) signaling pathway
*VRZAG29* ^a^	*5,286,106*		Protein TIFY 7	(chr1:5284375..5286232)	Regulation of flower development, jasmonate-regulated plant fertility
GF01-02_117	5,578,059	23.775	Transcription factor	(chr1:5581602..5585183)	Positive regulation of flower development
			Two-component response regulator ARR2	(chr1:5599937..5604835)	Cytokinin and ethylene mediated signaling pathway
			Protein POLLEN DEFECTIVE IN GUIDANCE 1	(chr1:5836691..5846071)	Required for micropylar pollen tube guidance
			PHD finger protein MALE STERILITY 1	(chr1:6187995..6190572)	Transcriptional activator required for anther and post-meiotic pollen development and maturation. Seems to regulate inflorescence branching and floral development
GF01-21	6,230,506	25.817	Putative Phytosulfokine receptor	(chr1:6227286..6230564)	Positive regulation of flower development
			STA1-12 (Tasselseed2)	(chr1:6584849..6585616)	Oxidoreductase activity; masculinizing; male-specific gene expression

Indicated are marker name, its physical location and position in cM on the genetic map as well as predicted candidate genes, their proposed functions and physical location as listed in the 12x reference genome (http://www.genoscope.cns.fr/externe/GenomeBrowser/Vitis/)

^a^In italics: Corresponding marker in the second mapping population GF.GA-47-42 × ‘Villard Blanc’

**Table 6:** Selected candidate genes annotated in the reference genome sequence of PN40024 for the flowering time QTL around marker GF01-22 on chromosome 1

Marker	Physical location	Location in cM	Candidate gene	Location of gene on physical map	Proposed function(s)
			Similarity to the auxin-independent growth promoter	(chr1:7122286..7137218)	Plant growth and development
GF01-22	7,253,589	30.613	Protein phosphatase 2C 14	(chr1:7249320..7253569)	Signal transduction, ABA signaling
			RING/U-box domain-containing protein, zinc finger	(chr1:7278912..7279343)	Photoperiodism, flowering
			Putative RPT2	(chr1:7324477..7326887)	Flower development
			Homeobox protein BEL1 homolog, KNOX	(chr1:7552421..7562329)	Plays a major role in ovule patterning and in determination of integument identity via its interaction with MADS-box factors
			Probable nucleoredoxin 1	(chr1:7894096..7907520)	Probable thiol-disulfide oxidoreductase required for pollen tube growth and pollen function in the pistil
			Ripening-induced protein	(chr1:8084005..8085831)	
			BHLH transcription factor	(chr1:8215768..8218154)	Transcription factor ABORTED MICROSPORES in *A. thaliana*; transcription factor. Plays a crucial role in tapetum development. Required for male fertility and pollen differentiation, especially during the post-meiotic transcriptional regulation of microspore development within the developing anther
GF01_32_87	8,243,137	33.169			
			Protein OVERLY TOLERANT TO SALT 1	(chr1:8712502..8728565)	Protease that catalyzes two essential functions in the SUMO pathway; Regulates salt stress responses and flowering time

Indicated are marker name, its physical location and position in cM on the genetic map as well as predicted candidate genes, their proposed functions and physical location as listed in the 12x reference genome (http://www.genoscope.cns.fr/externe/GenomeBrowser/Vitis/)

During further development of the flowers, the highest LOD value of the QTL switches slightly to a region further downstream around markers GF01-19/GF01-20 (Fig. 6). This region can also be found in the second mapping population with a QTL for the same trait. Within the associated 1-LOD confidence interval, several other genes are annotated that might also have an impact on flowering time and flower development. However, the most interesting candidates are probably two CONSTANS-like genes, situated within the LOD ± 1 confidence interval near markers VMC4f9.2 and GF01-19, respectively. In addition to several other zinc finger transcription factors, CONSTANS-like genes are known to play a key role in the control of flowering via the photoperiodic pathway in *A. thaliana* (Putterill et al. 1995). Two CONSTANS homologous genes have yet been identified in grapevine (VvCO and VvCOL1) and their expression patterns characterized (Almada et al. 2009). A spatial and temporal relationship in the expression of VvCO, VvFY and VvMADS8 (equivalent to the *Arabidopsis* genes LEAFY and SOC1) was observed during bud development. Taking into account that the respective QTL region is found in two genetically highly diverse mapping populations, an influence of the two CONSTANS-like genes on flowering time near the QTL peaks identified in this study seems very likely.

The gene *VvGAI1* (gibberellic acid-insensitive mutant protein 1) is located in the close proximity of the QTL peak, showing 63.5 % identity (76.5 % positives) to the *RGL2* gene from *A. thaliana*. Grapevine plants with a mutation in this gene have an altered response to gibberellic acid causing a dwarf habitus and the production of numerous inflorescences while normal tendril development is suppressed (Boss and Thomas 2002). Reversely speaking, one of the functions of gibberellic acid signals in grapevine seems to be the suppression of floral meristem production. This gene therefore represents another candidate gene for the control of the onset of flowering in grapevine.

Near the second QTL peak around marker GF01-22 (Fig. 6), several other annotated genes are present in the reference genome sequence that might function in flower development or flowering time control. These include an auxin-independent growth promoter, the gene for protein *SPIRAL1*-*like 2*, *RPT2* and the gene encoding the homeobox protein BEL1 homolog KNOX (Fig. 6). Several transporter protein genes and genes for non-defined transcription factors can be assigned to this region (Tables 5, 6). However, in this region well-known flowering time control genes like CO or FT do not appear in the reference genome sequence and we could not confirm this locus in the second mapping population. Further studies will thus be necessary to confirm whether these or other yet unknown genes contribute to regulation of flowering and ripening time in *Vitis*.

This study presents comprehensive data to develop new markers linked to phenological traits in marker-assisted breeding. We demonstrated that due to high synteny within the genus *Vitis*, it is easily possible to develop markers for fine mapping of specific loci. This relies on the availability of a high-quality reference genome sequence that allows a detailed deduction of candidate genes. Several of the QTLs for flowering time presented in this study were indentified using two independent mapping populations, permitting to identify functional candidate genes to be tested in the near future. A next step will be to unravel in more detail the pathways of phenological appearance in the field, a process that still is a challenge when addressing a highly complex perennial species such as grapevine. Our strategy proved suitable for the development of markers for extended marker-assisted selection, thus opening up new possibilities for fast and efficient modern grapevine breeding.

#### **Author contributions**

MD and IF carried out the phenotyping and genotyping steps. LH developed the concept of marker design based on the reference genome sequence. IF designed and carried out the data evaluation, construction of genetic maps for V3125 × ‘Börner’ and subsequent QTL analyses, DH and IF analyzed the sequences regarding candidate gene prediction. EZ provided the genetic map for GF.GA-47-42 × ‘Villard blanc’. LH, BW and RT designed the project and helped discuss and guide through all steps of the experiments. All the authors have read and approved the final manuscript.

## Electronic supplementary material

Below is the link to the electronic supplementary material.

Supplementary material 1 (XLS 118 kb)
